# Hydrogen peroxide-producing pyruvate oxidase from *Lactobacillus delbrueckii* is catalytically activated by phosphotidylethanolamine

**DOI:** 10.1186/s12866-020-01788-6

**Published:** 2020-05-24

**Authors:** Louis P. Cornacchione, Linden T. Hu

**Affiliations:** Department of Molecular Biology and Microbiology, Tufts University School of Medicine, Boston, MA 02111 USA

**Keywords:** Catalytic activation, Hydrogen peroxide, *Lactobacillus delbrueckii*, Pyruvate oxidase, Phospholipids

## Abstract

**Background:**

Pyruvate oxidase (Pox) is an important enzyme in bacterial metabolism for increasing ATP production and providing a fitness advantage via hydrogen peroxide production. However, few Pox enzymes have been characterized from bacterial species. The tetrameric non-hydrogen-peroxide producing Pox from *E. coli* is activated by phospholipids, which is important for its function in vivo.

**Results:**

We characterized the hydrogenperoxide-producing Pox from *L. delbrueckii* strain STYM1 and showed it is specifically activated by phosphotidylethanolamine (16:0–18:1), but not by phosphotidylcholine or phosphotidylglycerol. This activation is a mixture of K- and V-type activation as both k_m_ and enzyme turnover are altered. Furthermore, we demonstrated that the *L. delbrueckii* Pox forms pentamers and either decamers or dimers of pentamers in solution, which is different from other characterized Pox enzymes. Lastly, we generated a C-terminal truncation mutant that was only weakly activated by phosphotidylethanolamine, which suggests the C-terminus is important for lipid activation.

**Conclusions:**

To our knowledge this is the first known hydrogenperoxide-producing Pox enzyme that is activated by phospholipids. Our results suggest that there are substantial differences between Pox enzymes from different bacterial species, which could be important for their role in biological systems as well as in the development of Pox-based biosensors.

## Background

Pyruvate oxidase (Pox) is an enzyme in the oxidoreductase family. There are hydrogen peroxide-producing and acetate-producing Pox enzymes. In hydrogenperoxide-producing Pox enzymes, pyruvate, phosphate, and oxygen are converted to acetylphosphate, carbon dioxide, and hydrogen peroxide with the cofactors flavin adenine dinucleotide (FAD) and thiamine pyrophosphate (TPP) [1]. Acetate-producing Pox enzymes catalyze the oxidative decarboxylation of pyruvate to form carbon dioxide and acetate with electrons being transferred directly to the electron transport chain via the membrane-embedded electron carriers [2, 3].

The most well studied acetate-producing Pox is from *Escherichia coli*. *E. coli* Pox is dependent on the same cofactors as other Pox enzymes and is also homo-tetrameric [4]. *E. coli* Pox produces acetate and does not utilize oxygen as its final electron acceptor, but rather the membrane embedded ubiquinone 8 electron transport molecule [2]. CidC is another acetate-producing Pox enzyme from *Staphylococcus aureus* which transfers electrons to menaquinone and has an important role in cell death pathways [3]. Pyruvate:Quinone Oxidoreductase (PQO) from *Corynebacterium glutamicum* also produces acetate and transfers electrons to a quinone [5]. One characteristic of *E. coli* Pox, PQO, and CidC is that they are catalytically activated by phospholipids, which is thought to facilitate efficient transfer to membrane electron shuttles by activating the enzyme at a membrane peripheral position [3, 5, 6]. Mutants in *E. coli* Pox that disrupt lipid activation of the enzyme are localized to the C-terminal region of the protein. Further biochemical analysis identified a lipid activation helix in the C-terminal region (amino acids 558–568) that upon lipid interaction induce the preceding alpha helix to move out of the active site, which in turn positions phenylalanine 465 into the active site enhancing electron transfer between TPP and FAD [7–9]. The phospholipid activation of *E. coli* Pox is a hybrid of K_m_-driven (K-type) and velocity-driven (V-type) allosteric activation where the k_m_ for pyruvate decreases along with an increase in the enzyme turnover rate [10]. However, in CidC the activation is V-type where the enzyme turnover rate is substantially increased [3].

Hydrogen peroxide-generating Pox enzymes are produced by multiple different bacteria. Pox enzymes from *Lactobacillus plantarum* and *Streptococcus pneumoniae* are the most well studied. These enzymes form homo-tetrameric structures [11, 12]. The role of Pox enzymes in central metabolism is thought to be increasing ATP production in concert with acetate kinase during aerobic metabolism [13]. In *S. pneumoniae,* which has no tricarboxylic acid (TCA) cycle, additional ATP production from glucose is believed to come from this pathway [10]. *L. plantarum* Pox is considered to be an important component of the enhanced biomass that is observed in aerobic growth by generating acetyl phosphate as a substrate for acetate kinase [13]. In addition, the production of hydrogen peroxide from Pox could also be important on a community level. Since hydrogen peroxide is toxic to some bacteria, Pox activity could confer a fitness advantage to the producer organism. In *S. pneumoniae, pox-*deficient mutants display decreased virulence in a rat model of disease likely through decreased adhesion properties, but also potentially through decreased competition with commensal bacteria [14]. *Streptococcus gordonii* and *Streptococcus sanguinis* are known to produce enough hydrogen peroxide primarily through Pox to inhibit the oral pathogen *Streptococcus mutans* [15]. Recently, *Lactobacillus delbrueckii* strains harboring intact *pox* genes were shown to produce enough hydrogen peroxide to inhibit the growth of the oral pathogen *Porphyromonas gingivalis* [16]. The characterization of hydrogen peroxide-producing strains and their respective encoded hydrogen peroxide-producing enzymes could be beneficial in the development of novel treatments for diseases like periodontitis.

In the current study, we characterize a hydrogen peroxide-producing Pox enzyme from *L. delbrueckii* that is catalytically activated specifically by phosphotidylethanolamine, a common bacterial membrane component. This activation is largely dependent on the C-terminal region of the protein. Further, we showed that the *L. delbrueckii* Pox adopts a pentameric structure and potentially a dimer of pentamers, which is novel for Pox enzymes. These characteristics are unique for hydrogenperoxide-producing Pox enzymes and demonstrate substantial variability in the structure and function of Pox from different bacterial species.

## Results

### Characterization of L. delbrueckii pox

The *E. coli* Pox has been well studied, and it is known that this enzyme is catalytically activated by phospholipids [6, 7, 17]. In addition, CidC and PQO from *S. aureus* and *C. gluticaticum,* respectively, are other pyruvate oxidases that are lipid activated [3, 5]. Another well studied Pox enzyme is from *Lactobacillus plantarum* [1, 13, 18]. This enzyme has not been shown to be catalytically activated by phospholipids indicating that there is variability in the catalytic activation of the Pox enzymes. Amino acid identity between the enzymes ranges from 25.9% between *L. plantarum* Pox and PQO to 46.4% between *E. coli* Pox and PQO (Table 1). For DNA sequence identity, the identity ranges from 50.7% between CidC and PQO to 56.3% for CidC and *L. plantarum* Pox (Table 1).

**Table 1:** Amino acid and DNA percent identity of pyruvate oxidase enzymes. Shown is the amino acid and DNA sequence identity of pyruvate oxidase genes and proteins

Amino Acid Sequence Identity				
Enzyme	*E. coli* Pox	CidC	PQO	*L. plantarum* Pox
*L. delbrueckii* Pox	27.3%	33.1%	25.8%	37.8%
*E. coli* Pox		31.7%	46.4%	28.8%
CidC			31.3%	32.9%
PQO				25.9%
DNA Sequence Identity				
Enzyme	*E. coli* Pox	CidC	PQO	*L. plantarum* Pox
*L. delbrueckii* Pox	52.4%	53.8%	52.2%	55.7%
*E. coli* Pox		51.3%	55.7%	52.2%
CidC			50.7%	56.3%
PQO				52.2%

**Fig. 1:**
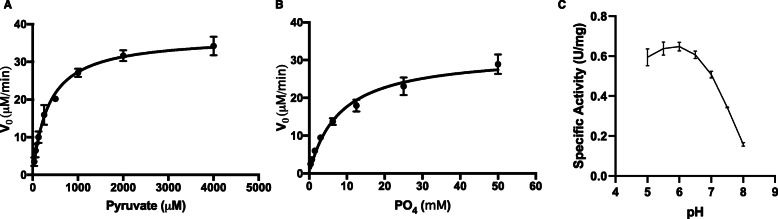
Kinetic parameters of *L. delbrueckii* Pox. **a**. Shown is the enzyme velocity of *L. delbrueckii* Pox at various concentrations of pyruvate in the reaction mixture. **b**. Shown is the enzyme velocity of *L. delbrueckii* Pox at various concentrations of phosphate in the reaction mixture. In both panels, enzyme concentration was 1.76 uM. The data represent the average of two independent experiments and error bars represent standard error. **c**. Shown is the enzyme specific activity at various pHs. The data represent the average of two independent experiments and error bars represent standard error

First, we wanted to determine the biochemical properties of the *L. delbrueckii* Pox enzyme. We previously purified *L. delbrueckii* Pox and used it for subsequent testing [16]. We determined the k_m_ values for pyruvate and phosphate and the k_cat_ value. The k_m_ for pyruvate and phosphate were 342.2 μM and 8 mM respectively, and the k_cat_ was 0.367 s^− 1^ (Fig. 1 and Table 2). The optimum pH for several other pyruvate oxidase enzymes is near 5.5 and so we tested the activity of the *L. delbrueckii* Pox at various pHs to determine its pH activity profile. In agreement with other Pox enzymes, the *L. delbrueckii* Pox was most active at the pH range of 5.5 to 6 (Fig. 1c).

**Table 2:** Kinetic parameters of *L. delbrueckii* Pox. Values for each parameter are listed with standard error

Km Pyruvate	Km PO4	Kcat
342.2 μM ± 44.9	8 mM ± 1.35	0.367 s-1 ± 0.013

### L. delbrueckii pox forms pentameric structure in solution

**Fig. 2:**
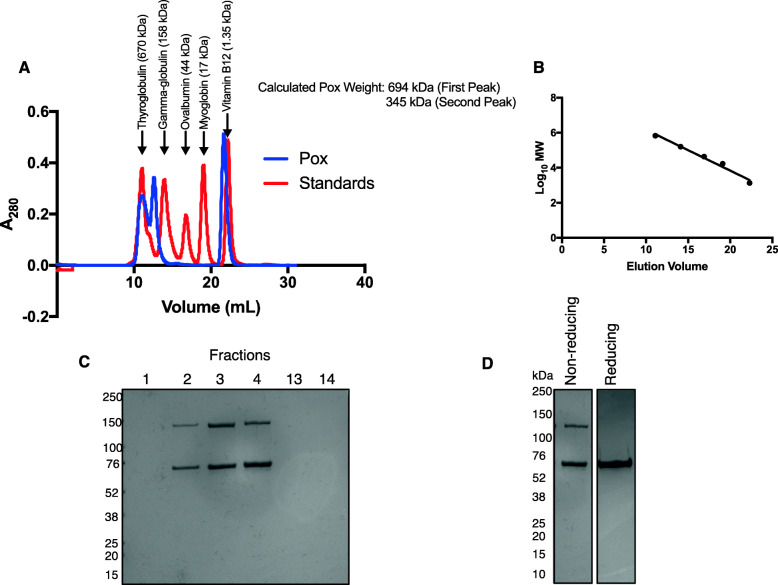
Oligomeric state of *L. delbrueckii* Pox. **a**. Size exclusion chromatography of *L*. *delbrueckii* Pox. 840 μg of Pox was combined with 50 mM pyruvate, 300uM TPP, and 15uM FAD and applied to the column. The absorbance at 280 nm is shown along with the elution position of known protein standards indicated by arrows. The calculated weight of the *L. delbrueckii* Pox is indicated based on the standard curve. 1 mL fractions were collected beginning at 10 mL elution volume to the end of the elution. **b**. Standard curve of gel filtration elution volumes for known protein standards. **c**. Coomassie stain of indicated fractions from gel filtration after SDS-PAGE under non-reducing conditions. **d**. Coomassie stain of purified *L. delbrueckii* Pox after SDS-PAGE under reducing (5% beta-mercaptoethanol) and non-reducing conditions

Both the *L. plantarum* and *E. coli* Pox are known to form tetrameric structures [1, 4]. To determine whether the *L. delbrueckii* Pox also formed a tetramer, we performed gel filtration chromatography with the purified enzyme. Interestingly, *L. delbrueckii* Pox eluted in two major peaks; the first with a calculated molecular weight of 694 kDa and the second with a calculated molecular weight of 345 kDa (Fig. 2a and b). The molecular weight of an STYM1 *L. delbrueckii* Pox monomer is 68 kDa according to the translation of the gene sequence; therefore, the first peak corresponded to a decamer and the second peak to a pentamer. The last peak corresponded to unbound FAD (Fig. 2a). Analysis of the fractions by non-reducing SDS-PAGE revealed two distinct bands that migrated distances consistent with monomer and dimer forms of the *L. delbrueckii* Pox at approximately 68 kDa and 136 kDa (Fig. 2c). Fractions 13 and 14, which correspond to the last peak from gel filtration, did not contain any protein and also were yellow in color confirming the presence of unbound FAD (Fig. 2c). To confirm that the 136 kDa protein band was in fact a dimer of *L. delbrueckii* Pox, we analyzed the *L. delbrueckii* Pox on non-reducing and a reducing SDS-PAGE. Under reducing conditions, *L. delbrueckii* Pox migrated exclusively as a monomer indicating that the 136 kDa band is a dimer, presumably linked by a disulfide bond at position 72 since it is the only cysteine in the amino acid sequence (Fig. 2d).

### Phospholipid activation of L. delbrueckii pox

**Fig. 3:**
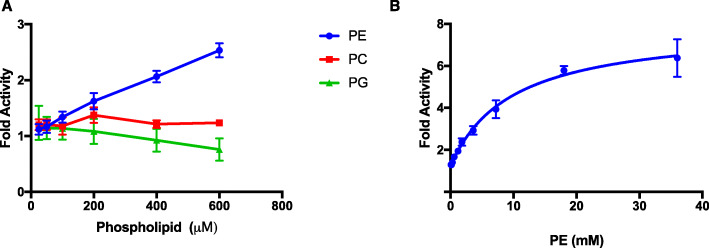
*L. delbrueckii* Pox is activated specifically by phosphotidylethanolamine. **a**. Shown is the fold activity of *L. delbrueckii* Pox relative to the enzyme activity with no added phospholipid with various concentrations of phosphotidylethanolamine (16:0–18:1) (PE), phosphotidylcholine (16:0–18:1) (PC), and phosphotidylglycerol (16:0–18:1) (PG). Data represent the average of two independent experiments and error bars represent the standard error. **b**. Shown is the fold activity of *L. delbrueckii* Pox with various concentrations of PE relative to enzyme activity with no PE. Data represent the average of at least two independent experiments and error bars represent the standard error

We hypothesized that *L. delbrueckii* Pox may be activated by phospholipids similar to *E. coli* Pox, CidC, and PQO [2, 3, 5]. We tested whether addition of either phosphotidylethanolamine (PE), phosphotidylcholine (PC), and phosphotidylglycerol (PG) could catalytically activate *L. delbrueckii* Pox. *L. delbrueckii* Pox was catalytically activated specifically by PE, but not PC or PG (Fig. 3a). The phospholipids used contained the same acyl chain structure (16:0–18:1) and only varied in the headgroup indicating that there is specificity in phospholipid activation of *L. delbrueckii*. To determine the full activation profile of *L. delbrueckii* Pox by PE, we measured the fold activity over a wider range of PE concentrations and observed activation upwards of 6-fold relative to the enzyme without PE (Fig. 3b). This level of activation exceeds that of the CidC Pox (3-fold activation) from *S. aureus* [3].

**Fig. 4:**
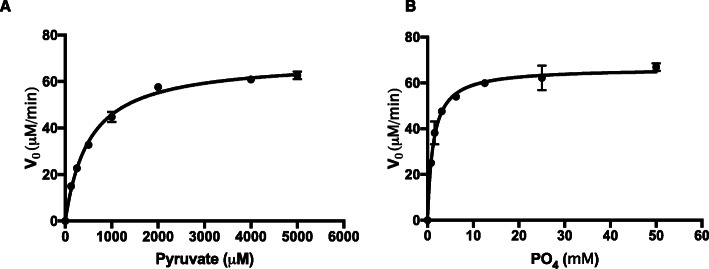
Kinetic parameters of *L. delbrueckii* in the presence of PE. **a**. Shown is the enzyme velocity of *L. delbrueckii* Pox at various concentrations of pyruvate in the reaction mixture with 7.2 mM PE. **b**. Shown is the enzyme velocity of *L. delbrueckii* Pox at various concentrations of phosphate in the reaction mixture with 7.2 mM PE. In both panels, enzyme concentration was 170 nM. The data represent the average of two independent experiments and error bars represent standard error

To gain mechanistic insight into how PE activates the enzyme, we tested the kinetic parameters of *L. delbrueckii* Pox in the presence of PE (Fig. 4). With the addition of PE, the k_m_ for pyruvate and phosphate were 519.9 μM and 1.25 mM respectively and the k_cat_ was 6.85 s^− 1^ (Table 3). The approximately 18-fold increase in k_cat_ and decrease in phosphate k_m_ relative to the enzyme in the absence of PE suggest that these parameters are the primary drivers of catalytic activation, which is consistent with the increase in k_cat_ observed in *E. coli* Pox and CidC upon phospholipid activation [3, 19]. The activation observed here is a hybrid of V- and K-type activation since the k_cat_ increased and the phosphate k_m_ decreased, which is also consistent with *E. coli* Pox [9, 19].

**Table 3:** Kinetic parameters of *L. delbrueckii* Pox with PE. Values For each parameter are listed with standard error

Km Pyruvate	Km PO4	Kcat
519.9 μM ± 31.85	1.25 mM ± 0.1336	6.85 s-1 ± 0.12

**Fig. 5:**
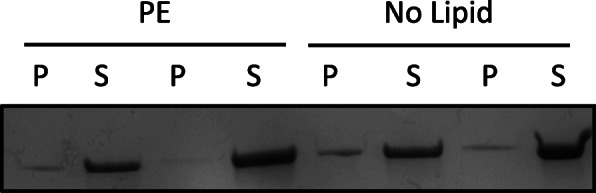
*L. delbrueckii* does not co-precipitate with PE. *L. delbrueckii* Pox with 50 mM pyruvate, 300uM TPP, and 15uM FAD was incubated with 1 mM PE or no lipid. The lipid fraction was pelleted by centrifugation after 30 min. Shown is a Coomassie stain of either the lipid pellet fraction (P) or the soluble fraction (S)

We also wanted to determine whether *L. delbrueckii* Pox inserts into PE micelles. We performed co-precipitation experiments with *L. delbrueckii* Pox in the presence and absence of PE. Nearly all of the enzyme remained in the soluble fraction and did not co-precipitate with PE, which is in contrast to *E. coli* Pox [7] (Fig. 5).

### The C-terminus of L. delbrueckii pox is important for lipid activation

**Fig. 6:**
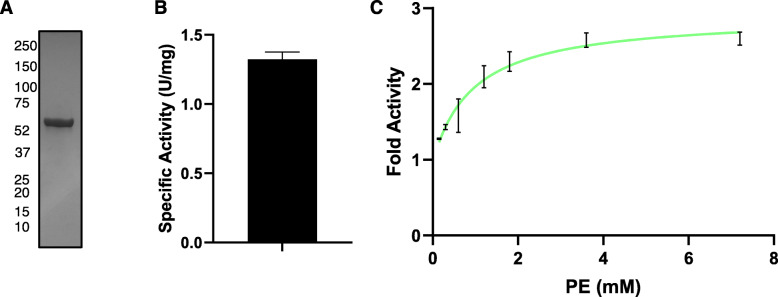
The C-terminal 34 amino acids are important for lipid activation. **a**. Coomassie stain of purified Δ34 Pox enzyme. **b**. Specific activity of the purified Δ34 Pox enzyme. Data represent the average of two independent experiments and the error bar represent standard error. **c**. Shown is the fold activity of Δ34 Pox enzyme with various concentrations of PE relative to enzyme activity with no PE. Data represent the average of two independent experiments and error bars represent the standard error

The C-terminus of *E. coli* Pox is important for lipid activation of the enzyme [7]. To test if the C-terminus was important in the *L. delbrueckii* Pox, we generated a C-terminal truncation that lacked the last 34 amino acids. We purified the enzyme and tested it for its ability to be activated by PE (Fig. 6a). The enzyme had a specific activity of approximately 1.32 U/mg in the absence of PE, which is greater than the specific activity of the full-length enzyme (Fig. 1c and Fig. 6b). This is consistent with the *E. coli* pox truncation having greater activity partially mimicking lipid activation [20]. Furthermore, the Δ34 enzyme was only weakly activated by PE to approximately 2-fold greater than the enzyme without PE compared to an approximately 6-fold activation with the full-length enzyme (Fig. 3b and Fig. 6c). This indicates that the C-terminal sequence is important for the lipid activation of the Pox enzyme.

## Discussion

Here, we demonstrated that the hydrogen peroxide-producing pyruvate oxidase enzyme from *L. delbrueckii* is catalytically activated specifically by PE, but not other phospholipids, and the last 34 amino acids of the enzyme are important for this activation. To our knowledge, this is the first hydrogenperoxide-producing Pox enzyme that has been shown to be activated by phospholipids. In addition, we showed that the *L. delbrueckii* Pox forms a pentameric structure in solution of which at least two subunits form a disulfide bond between cysteine 72. Other Pox enzymes form tetrameric structures, and there has been no evidence of disulfide bond formation indicating that the *L. delbrueckii* Pox has a different structure than other Pox enzymes [1, 4].

It is interesting that only PE had an impact on catalytic activity. This suggests that the size, shape, and charge of the phospholipid head group is important for catalytic activation. If the Pox enzyme is interacting with phospholipid that is already incorporated into the membrane, the head group would be exposed to the cytoplasm and be in the closest proximity to the enzyme. The interaction with the cytoplasm-facing ethanolamine likely occurs through the C-terminal 34 amino acids. Similar to the *E. coli* Pox, we hypothesize that this interaction induces a conformational shift in the enzyme that enhances electron transfer between FAD and TPP and decreases the phosphate k_m_. This interaction is likely mediated by electrostatic forces. In fact, it is believed that CidC interaction with phospholipids is driven primarily by electrostatic forces suggesting there is interaction with the headgroup [3]. Furthermore, PE is known to be an abundant component of bacterial membranes, whereas PC is more commonly a eukaryotic and mammalian membrane component [21, 22]. Thus, activation only by PE is reasonable since *L. delbrueckii* Pox is a bacterial enzyme. Enhanced hydrogen peroxide production by *L. delbrueckii* could be beneficial in a complex microbial community. In fact, hydrogen peroxide production by Pox has been shown to be important in the inhibition of dental pathogens like *P. gingivalis* [16].

The pentameric structure of *L. delbrueckii* Pox is different from what has been observed with *E. coli* Pox and *L. plantarum* Pox [1, 4]. Based on our gel filtration data, the *L. delbrueckii* also forms a decamer or a dimer of pentamers, but it is unclear which of these may be occurring. Interestingly, we also observed formation of dimers linked by a disulfide bond which are a component of the pentameric structure since they were present in fractions with an elution volume consistent with pentameric molecular weight. Therefore, the structure of the pentamer must be comprised of either three monomers and one dimer or one monomer and two dimers.

*E. coli* Pox lipid activation is a mixture of K- and V-type allosteric activation where the pyruvate K_m_ decreases and the k_cat_ increases [19, 23]. The lipid activation observed with *L. delbrueckii* Pox was also a mixture of K- and V-type allosteric activation, however, the specific parameters that changed were different. The pyruvate k_m_ increased slightly while the phosphate k_m_ decreased. The K_cat_ did increase substantially (18-fold) similar to the *E. coli* Pox. Since the changes in pyruvate and phosphate k_m_ could offset one another, we believe that the lipid activation of *L. delbrueckii* Pox is primarily driven by increasing the enzyme turnover rate. This is consistent with structural data of *E. coli* Pox demonstrating that lipid activation’s primary effect on the enzyme is to shift a phenylalanine closer to the interface between TPP and FAD to increase the efficiency of electron transfer [9]. This is thought to increase the enzyme turnover rate substantially. It is also in agreement with the activation of CidC where the activation of the enzyme was exclusively driven by a 10-fold increase in k_cat_ [3].

*E. coli* Pox, CidC, and PQO are the only known lipid-activated Pox enzymes [3, 5, 7]. These Pox enzymes likely evolved this ability so that they are most active near or at the membrane where it can readily transfer electrons to a membrane electron shuttle [3, 9]. For this reason, *E. coli* Pox inserts and co-precipitates with lipids in the presence of substrate [7]. Likewise, the CidC pyruvate oxidase from *S. aureus* interacts with phospholipids, but the interaction is believed to be mediated by electrostatic forces indicating that there does not necessarily need to be insertion into the membrane [3]. We did not observe any co-precipitation of *L. delbrueckii* Pox with PE suggesting that there is no insertion or that the interaction may be unable to withstand centrifugal forces. The fact that *L. delbrueckii Pox* does not strongly associate with or insert into the membrane may be a mechanism to regulate hydrogen peroxide production. If the association were too strong or there was insertion into the membrane, excessive hydrogen peroxide production may intoxicate the cell.

*L. delbrueckii* Pox does not need to interact with the membrane since it does not transfer electrons to a membrane electron shuttle, but rather to oxygen. Therefore, the utility of lipid activation of *L. delbrueckii* Pox is less clear than it is for other pyruvate oxidase enzymes that transfer electrons to membrane-bound carriers. One hypothesis is that the lipid activation of a hydrogen peroxide-producing Pox could be beneficial because it would position hydrogen peroxide production at a peripheral point of the cell. Passive diffusion of hydrogen peroxide from this point would subject the interior of the cell to less hydrogen peroxide and excrete more through the membrane than if the enzyme were positioned in the middle of the cell. Thus, the lipid activation of the Pox enzyme in this case may reduce the self-toxicity of hydrogen peroxide production by *L. delbrueckii* by activating the enzyme only at the edges of the cell*.*

## Conclusions

In this report, we describe the first hydrogen peroxide-producing Pox enzyme that is activated by the phospholipid phosphotidylethanolamine. We also demonstrated a different oligomeric structure than other known Pox enzymes. Together, these data demonstrate that Pox enzymes from different bacterial species can vary in their structure and function. As Pox is an important enzyme in mediating bacterial interactions and microbial community structure, enhancing pathogenicity, and for certain biosensor applications, further understanding of the biochemical characteristics of Pox are warranted.

## Methods

### Pyruvate oxidase

Pyruvate oxidase enzyme or pyruvate:oxygen 2-oxidoreductase (phosphorylating) (EC 1.2.3.3) was derived from *Lactobacillus delbrueckii* STYM1 strain [16]. The pyruvate oxidase enzyme was purified as previously described [16].

### Purification of L. delbrueckii pox Δ34

The *L. delbrueckii pox* deletion mutant was PCR amplified from STYM1 genomic DNA using the primers listed in Table 4. The PCR product was ligated into the pFLAG-CTC vector (Sigma, St. Louis, MO) using the NdeI and XhoI (New England Biolabs, Ipswich, MA) restriction sites. The product was transformed into DH5α *E. coli* and the construct was confirmed by PCR and sequencing. The construct was transformed into LOBSTR *E. coli* (Kerafast, Boston, MA), in which the *L. delbrueckii* Δ34 Pox was expressed as a C-terminal His_6_-Pox. A starter culture was diluted 1:100 into 40 mL of LB supplemented with 100 μg/mL of carbenicillin (Sigma, St. Louis, MO) and grown at 37 °C until the OD_600_ reached 0.4–0.8. The cells were cooled on ice for 10 min and 1 mM isopropyl-β-D-thiogalactopyranoside (IPTG) (Sigma, St. Louis, MO) was added and the cells incubated at 25 °C with shaking for 20 h. The cells were harvested by centrifugation at 3200 x g for 20 min at 4 °C and resuspended in 1 mL 20 mM Bis-Tris 150 mM NaCl pH 7 with EDTA-free cOmplete protease inhibitor cocktail (Roche, Basel, Switzerland). The cells were lysed by sonication on ice and the soluble cell lysate was harvested by centrifugation at 16000 x g for 15 min at 4 °C. The soluble cell lysate was filtered through a 0.22 μm polyvinylidene fluoride (PVDF) filter and applied to a 1 mL bed volume of HisPure Ni-NTA resin (Thermofisher, Waltham, MA). The column was washed twice with two column volumes of buffer containing 25 mM imidazole (Fisher, Pittsburgh, PA) and the bound protein was eluted from the column in buffer containing 250 mM imidazole. Protein purity was assessed by SDS-PAGE and Coomassie staining.

**Table 4:** Primers used in this study

Primer	Description or purpose	Sequence
Pox_F_NdeI	Cloning of Δ34*pox* into pFLAG-CTC	AGATATCATATGGCAAAAATTAAGGGCGCAAAC
Pox_R_Δ34_XhoI	Cloning of Δ34*pox* into pFLAG-CTC, includes His_6_-tag and stop codon	AATTCCCTCGAGTTAGTGATGGTGATGGTGATGACTTCCTGCACCTGAAGCCGCGTCAATTTC

### Pyruvate oxidase activity assay and kinetic parameters

Pyruvate oxidase activity was measured as previously described [16]. Briefly, 50 mM sodium pyruvate, 15 μM FAD (Sigma, St. Louis, MO), 300 μM TPP (Sigma, St. Louis, MO), 0.03% N-ethyl-N-(2-hydroxy-3-sulfopropyl)-m-toluidine (Sigma, St. Louis, MO), 0.015% 4-aminoantipyrine (Sigma, St. Louis, MO), and 33 μg/mL horseradish peroxidase (Sigma, St. Louis, MO) were combined with purified pox enzyme. After a 10-min incubation at room temperature, sodium phosphate pH 5.6 was added to 50 mM final concentration to initiate the reaction. The formation of a quinoneimine dye at 25 °C was measured by an increase in absorbance at 550 nm and the specific activity was calculated as described. 1 unit of Pox activity is defined as the production of 1 μmol of hydrogen peroxide per minute and this was converted to μM/min for kinetic analysis.

For k_m_ determination of pyruvate and phosphate and determination of k_cat_, dilutions of substrate were used instead of the 50 mM concentration. Enzyme concentration was 1.76 μM. V_0_ for each substrate concentration was plotted and the data were fitted using Prism software (Graphpad, San Diego, CA) kcat non-linear regression function. For k_m_ and k_cat_ determination in the presence of phosphotidylethanolamine (PE), PE was used at a concentration of 7.2 mM and the enzyme concentration was 170 nM.

### Phospholipid activation assay

Phosphotidylethanolamine (16:0–18:1) (Avanti Lipids, Alabaster, AL), Phosphotidylcholine (16:0–18:1) (Avanti Lipids, Alabaster, AL), and Phosphotidylglycerol (16:0–18:1) (Avanti Lipids, Alabaster, AL) dissolved in chloroform were purchased commercially at a concentration of 10 mg/mL. The desired volume of 10 mg/mL phospholipid solution was evaporated under a steady stream of argon gas (Airgas, Radnor, PA) and dried in a vacuum desiccator for 1 h to evaporate residual chloroform and yield the desired weight of dried phospholipid. The phospholipids were resuspended in the appropriate volume of 20 mM Bis-Tris 150 mM NaCl pH 6 to achieve the desired concentration and incubated at 37 °C with shaking at 225 rpm for 30 min. The suspension was vortexed for 30 s and vortexed immediately prior to each use.

Phospholipids were added to the pyruvate oxidase assay mixture at various concentrations and enzyme activity was measured as described above. Fold activity was calculated relative to enzyme activity without phospholipid.

### Gel filtration analysis

Prior to analysis of the *L. delbrueckii* Pox, protein standards were mixed together and applied to the Superdex 200 column (GE healthcare, Chicago, IL) using a duo-flow system (Biorad, Hercules, CA). The protein standards were thyroglobulin (670 kDa), gamma-globulin (150 kDa), ovalbumin (44 kDa), myoglobulin (17 kDa), and vitamin B12 (1.35 kDa). Protein was eluted from the column using a mobile phase of 20 mM Bis-Tris pH 6150 mM NaCl at 0.5 mL/min flow rate. A standard curve was plotted using the peak elution volume for each standard versus the Log_10_ of the molecular weight in daltons. Eight hundred forty μg of purified *L. delbrueckii* Pox was incubated in the presence of 50 mM pyruvate, 300 μM TPP, and 15 μM FAD for 10 min at room temperature prior to application to the column. The absorbance at 280 nm was recorded over the elution. One mL fractions were collected after the void volume of 10 mL until the end of the elution. The peak volume for the Pox fractions was used to calculate the molecular weight according to the standard curve.

*L. delbrueckii* Pox in each fraction from gel filtration was visualized after non-reducing SDS-PAGE analysis with Coomassie staining. For reducing SDS-PAGE analysis, 5% β-mercaptoethanol (Sigma, St. Louis, MO) was included in the sample buffer.

### PE co-sedimentation

PE was prepared as described above for activation experiments. 3 μg of *L. delbrueckii* Pox was incubated with 50 mM pyruvate, 300 μM TPP, and 15 μM FAD with either 1 mM PE or no PE for 30 min at room temperature. The PE fraction was precipitated by centrifugation at 16,000 x g for 20 min at 4 °C. The supernatant was aspirated as the soluble fraction and the PE pellet was resuspened in an equal amount of buffer. The fractions were visualized by Coomassie staining after SDS-PAGE.

## Data Availability

All data generated or analyzed during this study are included in this published article. Raw data can be made available upon request from the corresponding author.

## Abbreviations

Pox: Pyruvate oxidase
FAD: Flavin adenine dinucleotide
TPP: Thiamine pyrophosphate
PQO: Pyruvate:quinone oxidoreductase
K-type: K_m_-driven
V-type: Velocity-driven
TCA: Tricarboxylic acid
PE: Phosphotidylethanolamine
PC: Phosphotidylcholine
PG: Phosphotidylglycerol
IPTG: Isopropyl-β-D-thiogalactopyranoside
PVDF: Polyvinylidene fluoride
